# Multiple Metabolites Derived from Mushrooms and Their Beneficial Effect on Alzheimer’s Diseases

**DOI:** 10.3390/nu15122758

**Published:** 2023-06-15

**Authors:** Zijian Tong, Guodong Chu, Chenmeng Wan, Qiaoyu Wang, Jialing Yang, Zhaoli Meng, Linna Du, Jing Yang, Hongxia Ma

**Affiliations:** 1 College of Life Science, Engineering Research Center of the Chinese Ministry of Education for Bioreactor and Pharmaceutical Development, Jilin Agricultural University, Changchun 130118, China; t1010605895@163.com (Z.T.); cgd0429@163.com (G.C.); 20211233@mails.jlau.edu.cn (C.W.); tt22bb@126.com (Q.W.); 15981102518@163.com (J.Y.); 2 Laboratory of Tumor Immunolgy, The First Hospital of Jilin University, Changchun 130061, China; mengzl@jlu.edu.cn

**Keywords:** mushrooms, Alzheimer’s disease (AD), neurodegenerative diseases (NDs), metabolites, oxidation

## Abstract

Mushrooms with edible and medicinal potential have received widespread attention because of their diverse biological functions, nutritional value, and delicious taste, which are closely related to their rich active components. To date, many bioactive substances have been identified and purified from mushrooms, including proteins, carbohydrates, phenols, and vitamins. More importantly, molecules derived from mushrooms show great potential to alleviate the pathological manifestations of Alzheimer’s disease (AD), which seriously affects the health of elderly people. Compared with current therapeutic strategies aimed at symptomatic improvement, it is particularly important to identify natural products from resource-rich mushrooms that can modify the progression of AD. This review summarizes recent investigations of multiple constituents (carbohydrates, peptides, phenols, etc.) isolated from mushrooms to combat AD. In addition, the underlying molecular mechanisms of mushroom metabolites against AD are discussed. The various mechanisms involved in the antiAD activities of mushroom metabolites include antioxidant and anti-neuroinflammatory effects, apoptosis inhibition, and stimulation of neurite outgrowth, etc. This information will facilitate the application of mushroom-derived products in the treatment of AD. However, isolation of new metabolites from multiple types of mushrooms and further in vivo exploration of the molecular mechanisms underlying their antiAD effect are still required.

## 1. Introduction

During the past two hundred years, as food quality, medical care, and lifestyles have continually improved, the elderly population has proportionally increased [1]. It is reported that 9% of the global population is older than 65, and this number is estimated to reach 17% by 2050 [2]. The health problems of elderly people cannot be ignored. Today, Alzheimer’s disease (AD) is one of the neurodegenerative diseases (NDs) with a high incidence rate; these are disorders characterized by progressive dysfunction and death of neurons that seriously affect the health of many elderly people [3,4,5]. It is reported that the number of patients suffering from NDs was approximately 50 million in 2019, and it is estimated that this number will continue to increase to 152 million by 2060 [6]. If no effective prevention and treatment strategy is proposed, the number of Americans over 65 years old with AD is estimated to increase from 6.5 million to 13.8 million between 2022 and 2060 [7]. Furthermore, it is worth noting that a systematic analysis of the widespread corona virus disease of 2019 (COVID-19) and AD has been recently carried out. An increasing amount of data reveal that Severe Acute Respiratory Syndrome Coronavirus 2 (SARS-CoV-2) infections seriously damage the central nervous system (CNS) of patients, and this may further increase the incidence rate and severity of AD [8,9]. It is evident that AD is closely related to disability, motor dysfunction, dementia, and even death, thus representing a huge challenge, in particular for the elderly [10,11,12].

In recent years, as technology has developed and improved, many drug development and innovative treatment strategies for AD have emerged. However, although great efforts have been made to treat this disease, the currently available medical measures and medicines to halt its progression remain a source of frustration [10,13]. The majority of medications are targeted at the transient relief of the symptoms of AD rather than the underlying source of the disease [14]. Therefore, research on the prevention and treatment of AD is considered a global health issue. In this regard, natural functional ingredients isolated from mushrooms have been highlighted as potential innovative drugs [15,16].

Mushrooms are macroscopic fungi belonging to the *Basidiomycota* or *Ascomycota* phyla [17]. Historically, many wild mushrooms, such as *Ganoderma lucidum* and *Cordyceps*, have a wide range of edible and medicinal potential because of their diverse biological functions, nutritional value, and delicious taste, and these applications can be traced back to prehistoric times, particularly in Asian cultures [18,19,20,21,22]. Although some mushrooms have been found to be toxic, the beneficial activities of many mushrooms, including their antimicrobial, anticancer, and antioxidant properties, have been widely studied [23,24]. Additionally, mushroom resources are abundant in the world. Today, there may be as many as 140,000–160,000 mushroom species, and these diverse resources mean that mushrooms have great application potential in food, medicine, cosmetics, and other fields. However, to date, only a few species of mushrooms have been explored, accounting for only about 10% of all types of mushrooms, and of these, about 700 mushroom species have been found to be beneficial in the treatment of diseases [24,25]. Thus, there remains a large number of mushroom resources requiring further investigation.

**Figure 1:**
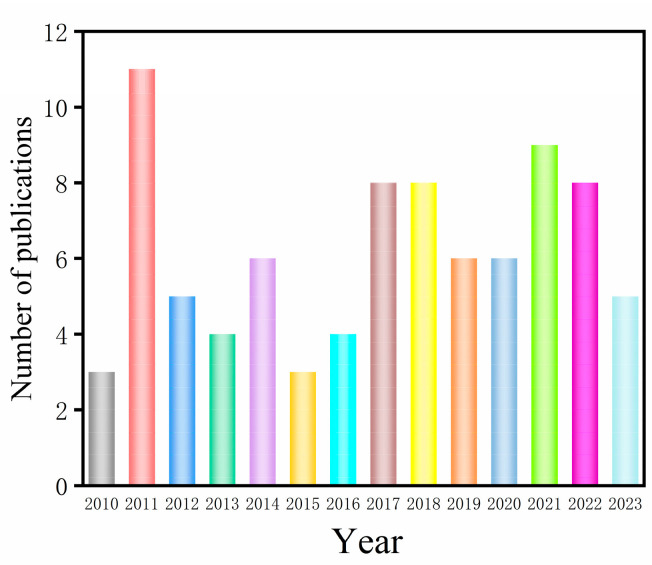
The number of publications between 2010 and 2023 covering the potential of mushrooms to alleviate AD.

Meanwhile, the number of articles recording the effects and molecular mechanisms of mushrooms in the treatment of NDs, and AD in particular, has gradually increased. The number of publications between 2010 and 2023 covering the potential of mushrooms to alleviate AD is shown in Figure 1. Including 2010, overall, about 86 research articles were found from the searches of the ScienceDirect database using the keywords “mushrooms” and “Alzheimer’s disease”. This review aims to summarize the beneficial roles of various mushroom metabolites for neuroprotection revealed in recent years, with an emphasis on AD.

## 2. The Main Targets for Treating Alzheimer’s Disease

**Figure 2:**
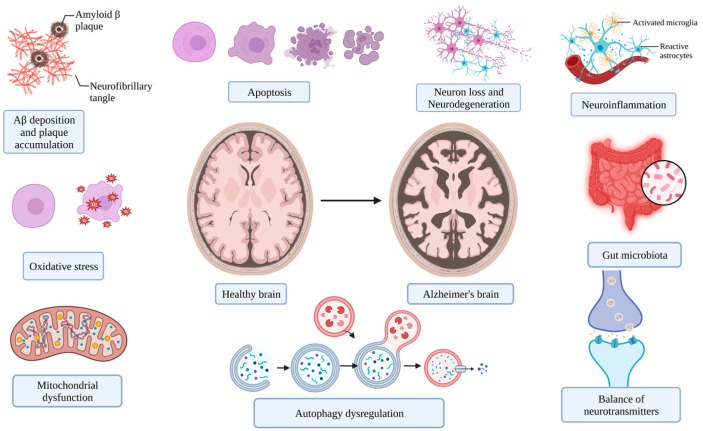
The main targets for the management of AD (figure was created with Biorender.com).

Analysis of pathophysiological mechanisms is crucial for selecting a novel drug for AD control. However, the etiology of this disease has unfortunately yet to be fully clarified, and this is a limiting factor in its prevention and control [26]. Nevertheless, the following cellular and molecular events associated with the occurrence and progression of AD are widely accepted (Figure 2) [13,27].

### 2.1. Deposition of Aggregated Proteins

As essential biological macromolecules, the precise folding of proteins is particularly important for the execution of their function. Once the normal folding of proteins is broken, the sticky surface can be exposed and eventually aggregate into nonfunctional and toxic fibers [28]. In terms of pathological changes, neurotic plaques formed by amyloid beta peptide (Aβ) and neurofibrillary tangles (NFTs) composed of hyperphosphorylated tau proteins are widely found in AD patients, which ultimately induce the loss of neurons as well as synapses in patients [29,30]. The amyloid fibrils or toxic oligomers aggregated by misfolded proteins (prion protein, TAR DNA-binding protein, microtubule-associated protein tau, Aβ, etc.) are deposited in specific neuron cells or tissues to form insoluble extracellular plaques and intracellular inclusions [31,32,33]. Misfolded or aggregated proteins increase the risk of AD because they cause protein dysfunction and even enhance toxic functions, which further leads to a reduction in neurons [34,35,36]. Aβ can cause multiple toxic reactions, such as the disruption of the intracellular calcium balance, abnormal membrane potential, and acceleration of cell apoptosis and synaptic loss [37]. An increasing number of attempts have been made to prevent the formation of protein aggregates, in addition to their elimination and regulation [38].

### 2.2. Modulation of Oxidative Stress

Once the balance between the generation and detoxified ability of oxidants is broken, oxidative stress is formed, which is usually characterized by an obvious increase in free radicals such as reactive oxygen species (ROS) and reactive nitrogen species (RNS) [39,40,41]. Generally, appropriate concentrations of ROS and RNS are particularly important for maintaining the normal function of living organisms as secondary messengers and can be scavenged by endogenous antioxidant systems, such as superoxide dismutase (SOD), glutathione peroxidase (GSH-Px), etc. [39,42,43,44,45]. However, oxidative stress can cause interference with the cell membrane function, irreversible damage to various functional cell components, including neuronal cell components (proteins, lipids, DNA, etc.), and even cell death. Additionally, there are reports concerning the deleterious effects of RNS on neurons [39,46,47]. Hence, oxidative stress undoubtedly poses a certain threat to many diseases (cardiovascular disease, diabetes, cancer, etc.) [48,49,50]. Because of its high oxygen demand, low antioxidant capacity, and large quantity of oxidizable unsaturated fatty acids, brain tissue is extremely susceptible to oxidative damage [51]. There is a significant amount of evidence to indicate that elevated oxidative stress is also closely associated with events including neuronal injury, aging and apoptosis, and inflammatory response, further accelerating the pathological changes and cell demise associated with AD and many other NDs [52,53]. Some studies have shown that Aβ plaque is able to reduce mitochondrial redox activity and further trigger ROS accumulation. Others consider that ROS contributes to the accumulation of Aβ in individuals with AD [54,55,56]. These findings support antioxidants as popular screening targets for antiAD drugs.

### 2.3. Alleviation of Neuroinflammation

There is emerging evidence that neuroinflammation is an important early event in AD [57]. Neuroinflammation, the defensive response of CNS to a range of adverse stimuli such as infection, brain injury, and toxins, is a key protective strategy for the body [58,59]. However, high-level and persistent neuroinflammation also has negative effects on nervous tissue [60]. Two types of CNS glial cells, namely, microglia and astrocytes, are considered to characterize neuroinflammation [58]. Overactivated CNS glial cells can lead to the release of pro-inflammatory cytokines (interleukin (IL)-1β, IL-6, tumor necrosis factor (TNF)-α, etc.), superoxide, free radicals, and cytotoxic mediators (nitric oxide, etc.), which may further induce neuronal death and synaptic dysfunction, inhibit neurogenesis, and worsen CNS damage through different pathways [38,61,62]. Furthermore, the production and deposition of Aβ are promoted by the cytokines and activated microglia [63,64,65].

### 2.4. Mitochondrial Dysfunction

Sufficient energy is particularly important for maintaining the survival and excitability of neurons, which mainly depend on the appropriate function of mitochondria. Mitochondria are widely involved in different physiological processes, including cell respiration, metabolism, energy generation through oxidative phosphorylation, intracellular signaling, cell survival and death, etc., [66,67]. In addition, ROS are generated and accumulated as by-products of the synthesis of ATP with the help of mitochondria and further destroy a variety of molecules [67,68]. Modern research shows that the accumulation of mitochondrial DNA deficiencies and mutant proteins in mitochondria, the decline of mitochondrial membrane potential (MMP), and calcium influx disorder are significant changes in delayed NDs. These pathological changes contribute to the regulation of neurotransmission and reduction in the survival of neurons [67,69]. Impaired mitochondrial biogenesis and trafficking, dysfunctional electron transport chains, imbalances in the concentration of calcium ions, and altered mitochondrial dynamics are all considered to be important pathways that participate in the occurrence and development of AD [70,71]. As a result, it is recognized that improving mitochondrial function is a potential treatment strategy to alleviate AD.

### 2.5. Cell Apoptosis, Necrosis, and Autophagy

Apoptosis, necrosis, and autophagy are all reported to play roles in neuronal cell death [72]. With regard to apoptosis, there is increasing evidence to confirm that neural cells with typical characteristics of apoptosis (DNA fragmentation, chromatin condensation, etc.) are present in AD and other NDs [73,74]. Apoptosis, an important process of programmed cell death (PCD), is thought to be the pathway of neuron loss in all NDs [75,76]. For example, overexpression of proapoptotic protein and downregulated antiapoptotic protein were detected in the brains of individuals with AD [73]. In addition, necrosis and autophagy are also reported to play critical roles in AD. Necrosis is mainly mediated by RIPK1 (receptor-interacting protein kinase 1), RIPK3 (receptor-interacting protein kinase 3), and MLKL (mixed lineage kinase domain-like protein), which form a complex called necrotic corpuscles, leading to necrosis [77]. It is reported that increased RIPK1 and MLKL levels are observed in AD patients. Additionally, it is widely accepted that autophagy as an intracellular degradation process is beneficial for eliminating misfolded proteins, and its relationship with NDs has received increasing attention [72]. It is also reported that a disorder of the autophagy process can promote the formation of Aβ plaque and NFTs, which further promote the release of proinflammatory factors, generation of ROS, and nerve cell death [78].

### 2.6. Other Factors

To date, excitotoxicity [79], dysregulation of neuronal calcium homeostasis [80], gut microbiota [81], cholinergic deficit heredity [82], and virus infection [83], etc. have also been reported to be related to NDs.

## 3. Diversity of Mushroom-Derived Metabolites Beneficial to AD and Their Possible Mechanisms

Many bioactive substances have been identified and purified from mushrooms, such as easily digested proteins, carbohydrates, terpenes, vitamins, etc. [84,85]. The diverse metabolites of mushrooms also demonstrate potential neuroprotective properties [86]. In this section, we will briefly discuss recent investigations and the possible mechanisms of important mushroom bioactive constituents for the alleviation of the pathological manifestations of AD.

### 3.1. Carbohydrates

**Figure 3:**
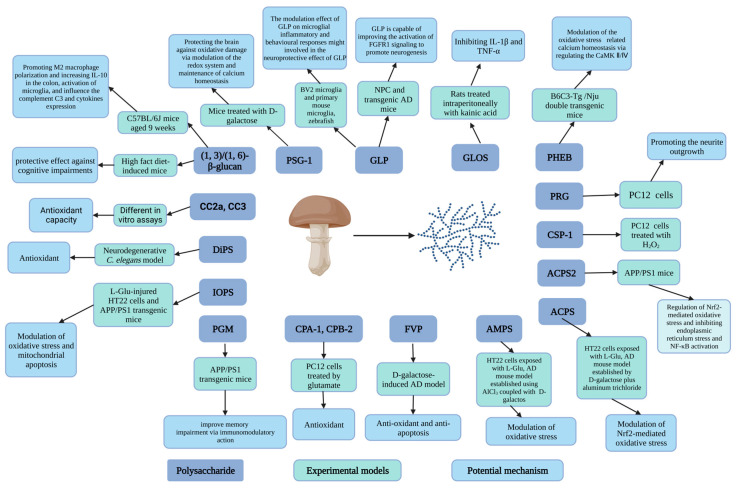
Schematic diagram of mushroom polysaccharides with antiAD effects and their mechanisms of action (figure was created with Biorender.com).

Carbohydrates, particularly polysaccharides, are the main nutrients in mushrooms. Polysaccharides, which are polymers composed of monosaccharides, have been found extensively in the hyphae of mushrooms [21]. Because of their advantages of low toxicity, biodegradability, stability, and low price, various polysaccharides derived from mushrooms have been increasingly valued for their neuroprotective properties [14,87,88]. To date, numerous researchers have provided a significant amount of evidence for the application of crude or purified polysaccharides in the treatment of AD. Typical polysaccharides and oligosaccharides isolated from mushrooms, together with their protective actions against AD, are listed in Table 1. Research has also been conducted to identify the neuroprotective mechanism of mushroom polysaccharides. To date, the neuroprotective mechanisms of mushroom polysaccharides have been primarily identified as the prevention of neuronal apoptosis [89] and oxidative damage [90], the reduction of Aβ deposition [91], the inhibition of acetylcholinesterase (AChE) [92], and the regulation of neuroinflammation [93] (Figure 3).

Polysaccharides extracted from *Dictyophora indusia*, *Pleurotus ostreatus*, and *Flammulina velutipes* have been proven to exhibit neuroprotective effects, which are related to their ability to alleviate the increase in ROS and peroxide products levels and enhance the activity of antioxidant enzymes [90,94,95]. *Amanita caesarea* is a nutritional mushroom widely grown in China, and the potential use of its isolated polysaccharides has been investigated. Li et al. purified polysaccharides called ACPS from a water extract of *A. caesarea*. They found that pretreatment of the hippocampal neuron cell line (HT22) with ACPS before exposure to _L_ glutamic acid (_L_ Glu) clearly improved the decline in cell viability, apoptosis rate, intracellular ROS level, and changes in MMP. Furthermore, improvements in abnormal behaviors were observed in AD model mice, together with reduced Aβ deposition and oxidative stress in the brain. They also found that this action of ACPS was attributed to its improvement of Nrf2-mediated oxidative stress [96]. Additionally, the polysaccharides from *A. caesarea* exhibit an anti-inflammatory effect and improve the cholinergic system function in vivo [97]. Similarly, *Hericium erinaceus* polysaccharide (HEP) reduced the neurotoxicity of pheochromocytoma cells (PC12) induced by _L_ Glu treatment, which was reported to be related to inducing cell differentiation, blocking overload of intracellular calcium, inhibiting ROS production, and preventing mitochondrial membrane depolarization. Furthermore, it was demonstrated that HEP improved memory impairment and behavior abnormality in mice with AD established by treatment with D-galactose and AlCl_3_ [98]. The study by Zhang et al. mentioned that a neutral polysaccharide (SCP-1) composed of a basic skeleton and branches was purified from an edible and medical mushroom, *Sparassis crispa*. It is noteworthy that the role of SCP-1 in the treatment of AD has also been observed in vivo. Using 16S rRNA sequencing technology, the growth of intestinal inflammation-related bacteria has been detected in AD mice treated with SCP-1, suggesting that this polysaccharide may alleviate the symptoms of AD by regulating intestinal microbiota and further inhibiting inflammation [99,100]. Additionally, as shown in Table 1, multiple polysaccharides isolated from various mushrooms, including *Grifola frondosa* [101], *G. lucidum* [102], *Armillaria mellea* [103], *Cordyceps cicadae* [104], *Pleurotus eryngii* [91], *Inonotus obliquus* [105], and *Tremella fuciformis* [106], are considered potential candidates for the management of AD.

In addition to polysaccharides, oligosaccharides also receive attention in terms of neuroprotection. Tello and coworkers attempted to assess the neuroprotective potential of the oligosaccharide fraction obtained from *G. lucidum*. They found that the changes in behavior and histopathology in rats treated with kainic acid were relieved [107]. Collectively, the above findings suggest that mushroom carbohydrates are promising candidates as therapeutic drugs for the treatment of AD.

**Table 1:** The studies of beneficial effect of mushroom carbohydrates on Alzheimer’s diseases in vivo and in vitro (Increase, ↑; Decrease, ↓).

Mushroom Species	Name	Molecule Weight (kDa)	Experimental Models	Dose and Periods	Effect	Potential Mechanism	Ref.
*Amanita caesarea*	Polysaccharide (ACPS)	18.620, 33.500	HT22 cells exposed to _L_-Glu, AD mouse model established by _D_-galactose plus aluminum trichloride	In vitro test: 2.5 or 5 μg/mL for 3 h. In vivo test: 2.5 or 5 mg/kg for 42 days	In vitro test: Cell viability, MMP ↑ Apoptotic rate, ROS levels, intracellular Ca^2+^ ↓ The expression of Bcl-2, HO-1, SOD1, GCLC and the Nrf2 levels in nucleus ↑ The expression of Bax, cleaved caspase-3, Keap-1, cytochrome C and the Nrf2 levels in cytoplasm ↓ In vivo test: AD-like behavior, Aβ1-42 level in brain, Aβ plaque, Ach and choline ChAT, SOD ↓ Aβ1-42 level in serum, AChE, GSH-Px, SOD ↑	Modulation of Nrf2-mediated oxidative stress	[96]
	Polysaccharide (ACPS2)	16.6	APP/PS1 mice	6 weeks	Cognition ability and anxious behavior ↑ Tumor necrosis factor-α, interleukin-1β ↓ Brain injury, Aβ deposition, tau hyperphosphorylation↓	Regulation of Nrf2-mediated oxidative stress and further inhibiting endoplasmic reticulum stress and nuclear factor-kappa B (NF-κB) activation	[97]
*Armillaria mellea*	Mycelium polysaccharides (AMPS)		HT22 cells exposed to _L_-Glu, AD mouse model established using AlCl_3_ coupled with D-galactos	In vitro test: 10, 20, 40, and 80 μg/mL for 3 h In vivo test: 25, 100 mg/kg/day, 4 weeks	In vitro test: Cell viability, Mitochondrial membrane potential (MMP) depolarization ↑ Nuclear apoptosis, ROS, Caspase-3 activity ↓ In vivo test: AD-like behavior, TUNEL-positive apoptotic neurons, AchE level, ROS, the expression of Aβ in the hippocampus, 4-NHE levels, and p-Tau aggregation ↓ Ach level, ChAT level, SOD and GSH-Px level, serum Aβ_1-42_ concentrations ↑	Modulation of oxidative stress and antiapoptosis	[103]
*Cantharellus cibarius*	Polysaccharide fractions (CC2a, CC3)		Different in vitro assays	10, 25, 50, 100 μg/mL, 48 h	Neurons viability and neurite outgrowth ↑ LDH level in cell culture medium ↓ Mitochondrial dehydrogenase activity ↑ Lactate dehydrogenase activity ↓ Neurite outgrowth ↑ DCF ↓	Antioxidant capacity	[108]
*Cordyceps cicadae*	Polysaccharides (CPA-1, CPB-2)		PC12 (pheochromocytoma) cells treated with glutamate	25, 50, 100, and 200 μg/mL, 24 h	Cell viability, GSH-Px activity, SOD activity ↑ LDH breakage, ROS production, intracellular Ca^2+^ level, MDA level ↓	Antioxidant	[104]
*Cordyceps sinensis*	Polysaccharide (CSP-1)	210	PC12 cells treated with H_2_O_2_	25, 50, 100 μg/mL	Survival of cells, the activity of SOD and GSH-P ↑ MDA level ↓		[109]
*Dictyophora indusiata*	Polysaccharides (DiPS)		Neurodegenerative *C*. *elegans* model	0.5–4.0 mg/mL, various times	Survival rate, SOD activity, mitochondrial membrane potential, and ATP content ↑ ROS and MDA levels ↓ DAF-16/FOXO ↑ polyQ- and Aβ-mediated behavior disorders ↓	Antioxidant	[90]
*Flammulina velutipes*	Polysaccharide (FVP)		D-galactose-induced AD model	400 mg/kg/d, 30 days	Cognitive ability ↑ SOD, CAT, and GSH-Px activities, Bcl-2 expression ↑ Apoptosis rate, Bax, cytochrome C, caspase-3, caspase-9, apoptosis-inducing factor expression levels, MDA level ↓	Anti-oxidant and anti-apoptosis	[95]
*Hericium erinaceus*	Polysaccharide (PHEB)	36.1	B6C3-Tg (APPswePSEN1d E9)/Nju double transgenic mice	25 and 100 mg/kg body weight, 6 weeks	Cognitive behavior, ChAT, and Ach level, serum levels of Aβ_1-42_, SOD and GSH-Px activity, the levels of Nrf2, the expression of mTOR, SHANK3, Akt, GABBR1, PKA, GluT1, Neurogranin ↑ Inflammation in brains, AChE, Aβ plaque area, phosphorylated tau plaques, and neurofibrillary tangles in hippocampus, MDA and ROS levels, the levels of Keap1 ↓ P-Ca2+/calmodulin-dependent kinase Ⅳ (CaMKⅣ), P-CaMK Ⅱ, ERK 1/2, Ras, P-GluR2 ↓	Modulation of the oxidative stress-related calcium homeostasis via regulating the CaMK Ⅱ/Ⅳ	[110]
*Ganoderma atrum*	Polysaccharide (PSG-1)	1013	Mice treated with D-galactose	50, 100, or 150 mg/kg body weight, 4 weeks, once a day	SOD, CAT, GPx, and GSH-Rd activities, GSH content ↑ GSSG and MDA level, apoptosis, ROS production, and calcium levels ↓	Protecting the brain against oxidative damage via modulation of the redox system and maintenance of calcium homeostasis	[111,112]
*Ganoderma lucidum*	Polysaccharide (GLP)	15	Neural progenitor cell (NPC) and transgenic AD mice	In vivo test: 30 mg/kg body weight, once per day, 90 days; In vitro test: 10, 30, 100, 300 μg/mL	Cognitive function ↑ Double-positive cells (BrdU/NeuN) number in the hippocampus ↑ The number of Ki67 and SOX2 double-positive proliferation NPC, Phosphorylation of FGFR1, ERK, AKT ↑ 6E10-postitive Aβ area ↓	GLP is capable of improving the activation of fibroblast growth factor receptor 1 (FGFR1) signaling to promote neurogenesis	[102]
			BV2 microglia and primary mouse microglia, zebrafish	In vitro assays: 2 h, 1–1000 ng/mL for BV2, 0.3–100 ng/mL for primary microglia In Zebrafish, 1 μg/mL, 12 h-5 d postfertilisation	IL-1β, IL-6 and iNOS expression ↓ The expression of TGFβ ↑ MCP-1 and C1q expressions ↓ Microglial migration, morphological alterations, and phagocytosis probabilities ↓	The modulation effect of GLP on microglial inflammatory and behavioral responses might be involved in the neuroprotective effect of GLP	[93]
	Oligosaccharide fraction (GLOS)	0.8–1.3	Rats treated intraperitoneally with kainic acid (10 mg/kg body weight)	10, 40, 80 mg/kg body weight	Mortality, neuronal loss, staining for (GFAP), the expression of IL-1β and TNF-α ↓	Inhibiting the production of glia-derived toxic factors (IL-1β and TNF-α)	[107]
*Grifola frondosa*	Proteo-β-glucan (PGM)		APPswe/PS1ΔE9 (APP/PS1) transgenic mice (AD model)	intraperitoneal injection of PGM (5, 10, 20 mg/kg body weight per day) for 3 months	Learning and memory capability, the number of Nissl bodies and neurons, the expression of astrocyte marker (GFAP) and microglial marker (Iba1), microglial recruitment to the Aβ plaques, Aβ phagocytosis ↑ Histopathological abnormalities and necrotic neurons, the mean area containing Aβ1-42-positive plaques ↓	PGM could improve memory impairment via immunomodulatory action	[101]
*Inonotus obliquus*	Polysaccharide (IOPS)	111.9	L-glutamic acid (L-Glu)-injured HT22 cells and amyloid precursor protein/presenilin 1 (APP/PS1) transgenic mice	In vitro test: 5 or 10 μg/mL for 3 h In vivo test: 25 or 50 mg/kg/d (i.g.), once daily, 8 weeks	In vitro test: Cell viability ↑ Apoptosis, caspase-3 activity, release of LDH, ROS, the levels of Bax and Keap1↓ MMP, Bcl-2, Nrf2, HO-1, SOD-1 and cysteine ligase catalytic subunit (GCLC) ↑ In vivo test: Memory and cognition ability ↑ Aβ1-42 deposition, the number of neuronal fiber tangles, 4-HNE, and Keap1 levels in brain↓ SOD and GSH-Px level, Nrf2, HO-1, GCLC and SOD-1↑	Modulation of oxidative stress and mitochondrial apoptosis	[105]
*Lentinula edodes*	(1, 3)/(1, 6)-β-glucan		High-fat diet-induced mice	Mice supplemented with β-glucan from I. edodes (500 mg/kg food) for 7 days or 15 weeks	The abundance of Proteobacteria, energy intake, the order Clostridiales, class Clostridia, family Lachnospiracease, and family Ruminococcaceae in mice short-term supplemented with β-glucan. ↑ The proportion of Firmicutes, Proteobacteria, Actinobacteria in mice long-term supplemented with β-glucan. ↓ Discrimination index, body weight ↑ Cognitive decline, serum LPS, macrophage marker F4/80 positive cells, the expression of IL-6, TNF-α and IL-1β, microglial number, the proliferation of microglia, the expression of BDNF and PSD-95 ↓ The expression of occludin ↑	The protective effect against cognitive impairments of sample was demonstrated via colon–brain axis improvement in mice induced by the HF diet	[113]
			C57BL/6J mice aged 9 weeks	60 mg/kg body weight, 15 weeks	The discrimination index, brain-derived neurotrophic factor (BDNF), the CD206+ cell number in colon, IL-10 expression ↑ The number of Ibal1 positive cells, the expression of complement C3, IL-6, IL-1 β and TNF-α ↓	Promoting M2 macrophage polarization and increasing IL-10 in the colon, activation of microglia, and influencing the complement C3 and cytokines expression	[114]
*Phellinus ribis*	Polysaccharide (PRG)	5.16	PC12 (pheochromocytoma) cells	10, 50, 150 μg/mL	Mean neurite lengths of NGF-stimulated PC12 cells ↑	Promoting the neurite outgrowth	[115]
*Pleurotus ostreatus*	Polysaccharide (POP)	24	D-galactose and AlCl3-induced AD rats	400 mg/kg body weight, 30 days	Learning and memory capability ↑ SOD, GSH-Px, and CAT activities in hippocampus, liver, and serum ↑ MDA level in hippocampus, liver, and serum and hippocampal AchE activity ↓ Protein phosphatase 2A (PP2A) ↑ The expression of amyloid precursor protein (APP), Aβ, β-site APP clearing enzyme1 (BACE1), p-tau, and glycogen synthase kinase 3beta (GSK-3β) ↓	Relieving the Aβ formation and tau phosphorylation	[94,116]
	Polysaccharide (POP-W)	3.034 × 10^3^	PC12 cells damaged by H_2_O_2_	0.1, 0.2, 0.4, 0.8, 1.6, 3.2 mg/mL, 24 h	Cell viability, SOD activity, GSH level, the ratio of Bcl-2/BAX, the p-Akt/Akt ratio, and PI3K expression ↑ LDH, MDA levels, Caspase-3 level ↓	POP-W pretreatment was able to protect PC12 cells against H_2_O_2_ damage due to its capacity of antioxidant and anti-apoptosis via regulating the PI3K/AKT signaling pathway and apoptosis-related pathway proteins	[117]
*Pleurotus eryngii*	Polysaccharide (PEP)		Aging rats and PC12 cells	In vitro test: 0.5, 1, 1.5 μM, 24 h. In vivo test: administered with PEP for 28 weeks	Cell viability ↑ Intracellular calcium, apoptosis, APP production in brain, iNOS, and COX-2 level ↓	Modulation of calcium channels or inflammation	[91]
*Pleurotus sajor-caju*	Polysaccharide (PSP2-1)	44.9	Neuronal cell HT22 induced by H_2_O_2_ and aging mice induced by D-galactose	In vitro test: 50, 100 to 150 μg/mL for 24 h In vivo test: 100, 200, and 400 mg/kg/d for 42 days	In vitro test: Cell viability, Mitochondrial membrane potential (MMP), and the expression ratio of bcl-2/bax ↑ LDH release and cytochrome c release, apoptosis rate, ROS level, and the expression of cleaved caspase-3, cleaved PARP, Erk1/2, JNK, p38 ↓ In vivo test: Learning and memory ability, CAT, and SOD ↑ MDA and ROS ↓	The protective actions of PSP2-1 on nerve cells against oxidative damage and apoptosis induced by hydrogen peroxide were attributed to its regulating the MAPK signaling pathway	[118]
*Sparassis crispa*	Polysaccharides (SCP-1)	13.68	C57BL/6J mice treated with D-galactose and AlCl_3_	25 and 100 mg/kg/d, 4 weeks	Learning and recognition, GABA and Ach levels in brain ↑ Aβ deposition and Aβ_1-42_, Glu ↓ IL-6, TNF-α, IL-1β, serum LPS ↓ Iba1-positive microglia and GFAP-positive astrocytes in hippocampal CA1 and DG area ↓ The expression of TLR4, NF-κB, and phosphorylation of NF-κB ↓ Altering the gut microbiota	Modulation of gut microbiota and suppression of inflammation	[100]
			HT22 cells treated by H_2_O_2_	10, 25, 50, 100, 200, 400, 800 μg/mL, 12 h	Cell viability, SOD, and GSH-Px activities ↑ ROS, MDA, chromatin condensation and apoptotic bodies, apoptotic rate ↓	Antioxidant and inhibiting apoptosis	[99]
*Tremella fuciformis*	Polysaccharide (TL04)	2.033	Glutamate-induced neurotoxicity in DPC12 cells	5 and 20 μg, 3 h	Cell viability ↑ LDH release, ROS, apoptotic nuclei ↓ Bcl-2 level and Cyto C level ↑ Bax expression, the levels of cleaved caspase-8, caspase-9 and caspase-3 ↓	The underlying mechanism for protective effect of TL04 against glutamate-induced neurotoxicity was proved to be associated with the caspase-dependent mitochondrial pathway	[106]

### 3.2. Proteins and Peptides

Because of their diverse biological functions, high affinity with specific targets, high membrane permeability, and many other special properties, peptides are considered important screening targets by the pharmaceutical industry [119,120]. The protein content in mushrooms is relatively high, with an average of approximately 23.80 ± 9.82 g of protein per 100 g of mushroom dry weight (d.w.) [121]. As a consequence, many researchers have sought to use a variety of mushroom peptide resources in the treatment of NDs. Furthermore, there is mounting evidence that natural bioactive peptides have great antioxidant and inflammatory inhibition potential, suggesting them as promising candidates for a role in neuroprotection [122,123]. For instance, the neuroprotective activity of protein hydrolysates obtained from *Pleurotus geesteranus* in H_2_O_2_-injured PC12 cells was investigated by Wu et al. The data indicated the presence of a large quantity of hydrophobic amino acids in the test hydrolysates, and samples exerted a clear neuroprotective effect by reducing the ROS level and improving the antioxidant properties [121]. Another peptide (10,906 Da) called cordymin was isolated from *Cordyces sinensis* and *Cordyceps militaris* [124,125]. This peptide was first noticed for its antifungal activity, and its neuroprotective potential was subsequently discovered. Research carried out by Wang et al. demonstrated that cordymin was capable of protecting against nerve damage in the ischemic brain through anti-inflammation and improved antioxidant capacity [124]. Additionally, Wu et al. demonstrated the neuroprotective action of two novel selenium peptides (Se-P1 and Se-P2) derived from selenium-enriched *C. militaris*, which was attributed to their ability to modulate oxidant stress, inflammation, and gut microflora [126].

The biosynthesis of ergothioneine, a thiol derivative of histidine found in many mushrooms, has been extensively described [127]. The accumulated evidence confirms the therapeutic activity of ergothioneine against various diseases [128,129]. More importantly, some studies have revealed that a low ergothioneine level in the blood is one serious threat factor for AD [130]. Wu et al. found that a low plasma ergothioneine level can be used to predict the decline of cognition in elderly subjects [131]. Furthermore, with the help of a unique transporter called organic cation transporter 1, ergothioneine can easily cross the blood–brain barrier, which is conducive to its activity. Recent studies have demonstrated that ergothioneine is effective in resisting the negative influences of neurotoxic substances on neurons and cognitive function [132,133]. The neuroprotective effect of ergothioneine was shown to be associated with its capabilities of antineuroinflammatory, antioxidant, neurogenesis promoting, and neurotrophic factor induction capabilities [134,135,136]. With regard to AD, the potential application of ergothioneine has been explored in different models. In a transgenic *Caenorhabditis elegans* overexpressing the human AD Aβ peptide, ergothioneine reduced Aβ deposition [137]. The protective activity of ergothioneine against AD was further assessed by Whitmore et al. In 5XFAD model mice administered with ergothioneine, they found that amyloid plaques and oxidative stress were markedly decreased, and glucose metabolism was enhanced [138]. Thus, dietary supplementation is a way to enhance ergothioneine levels and, thereby, improve the clinical symptoms of AD. Fortunately, some mushrooms are rich in ergothioneine with amounts of 0.21–2.60 mg/g d.w., a prerequisite for dietary supplementation with ergothioneine [127]. In summary, mushrooms are a valuable resource pool of peptides that can be used to target the pathological manifestations of AD.

### 3.3. Phenolic Compounds

A common nutrient in mushrooms, phenolic compounds, have received extensive attention recently. These compounds can be classified into multiple classes: flavonoids, phenolic acids, tannins, coumarins, etc. [139]. There is a large amount of evidence to suggest that mushroom phenolic compounds have a wide range of health benefits, including antitumor, antioxidant, and antimicrobial properties, which were reviewed by Abdelshfy et al. [140]. However, there has been little discussion of the protective ability of mushroom phenols against NDs. Modern research suggests that there are various phenolic substances in mushrooms. It is documented that flavonoids with the contents of 6.646, 6.854, and 9.187 mg quercetin/g were found in *Macrocybe gigantea* J124, *Lactifluus leptomerus* J201, and *Ramaria thindii* J470, respectively [141]. Additionally, 4-hydroxybenzoic acid, gentisic acid, and 4-coumaric acid were the main phenolic acids identified in *T. fuciformis*, and their contents were 323, 174, and 30 mg per kilogram of dried mushrooms, respectively [142]. The amount of total polyphenol in *P. ostreatus* was determined to be 487.12 mg gallic acid equivalent/100 g d.w. [143]. The fact that various mushroom phenolic compounds control the pathogenesis of neuronal disease has attracted the attention of many researchers. Ethanol extract from *Stereum hirsutum* was found to be potent in inhibiting AChE (enzyme hydrolysis, the key neurotransmitter acetylcholine), which may be thanks to the phenolics within it [144]. Hispidin, a polyphenolic nutrient identified from the medicinal mushrooms *Phellinus linteus* [145], *Phellinus igniarius* [146], *Phaeolus schweinitzii* [147], etc., was first taken as an example. In the past few years, there have been attempts to increase the yield of mushroom hispidin. In a study by Liang et al., after strain screening and culture process optimization, the maximum hispidin production per gram of mycelium by *P. linteus* 04 was increased to 1.107 mg [148]. To elucidate the biosynthetic mechanism of hispidin in *P. igniarius*, Guo et al. used iTRAQ proteomic analysis in their study [146]. It was found that hispidin was able to alleviate the neuroinflammation in BV-2 microglial cells induced by nitric oxide via ROS-dependent mitogen-activated protein kinase (MAPK) signaling [149]. Others demonstrated that hispidin from *Phellinus linteus* is capable of inhibiting β-secretase (BACE1) competitively, thereby reducing the formation of β-amyloid, amyloid precursor protein cleavage products [150].

In addition to hispidin, hericenones, which are phenolic derivatives identified in the fruiting bodies of *Hericium erinaceum* [151], also demonstrate neuroprotective capability. In recent years, the beneficial potential of *H. erinaceum* on NDs has been widely investigated. It was demonstrated that the expression level of the nerve growth factor (NGF) gene, secretion of NGF protein, and neurite outgrowth in 1321N1 human astrocytoma cells treated with ethanol extracts of *H. erinaceum* were all enhanced. Furthermore, enhanced expression of NGF was also detected in the hippocampus of ddY mice administrated with *H. erinaceum* extracts, and this was related to c-Jun N-terminal kinase (JNK) signaling [152]. After taking supplements containing *H. erinaceum* for 12 weeks, the cognitive functions of subjects undergoing clinical testing clearly improved [153]. In addition, during an investigation of isolating the metabolites of *H. erinaceum*, multiple hericenones and their analogs were obtained, such as hericenones C and D [154]. Phan and coworkers purified hericenones B–E from the basidiocarps of *H. erinaceum* and discovered that NGF secretion was stimulated by hericenone E via the MEK/ERK and PI3K/Akt pathways [155]. Additionally, it is widely known that protection against endoplasmic reticulum stress-induced apoptosis on neural cells is a key target for AD treatment. An analog of hericenone F, 3-hydroxyhericenone F purified from *H. erinaceum* was found to have a protective capacity against endoplasmic reticulum stress-dependent Neuro2a cell death [156].

A large number of phenols were also identified from mushrooms, such as gallic acid, ferulic acid, chlorogenic acid, caffeic acid, and anthraquinone [157]. Some of these plant-derived phenolic compounds were documented to have antiND potential [158,159,160,161,162]. Whether the administration of these phenolic compounds from mushrooms can also effectively alleviate the clinical symptoms of AD remains to be explored in vitro and in vivo.

### 3.4. Terpenes

Terpenes are another type of secondary metabolites of mushrooms which consist of isoprene repeating units, and many terpenes such as cyathane diterpenoids, triterpenoids, and sesquiterpenes have been identified or isolated from various genera of mushrooms [163,164,165]. Some of these terpenes have also been found to exhibit the potential to improve the pathology of NDs. For example, after the application of tritepenoids from *G. lucidum* for a long time, the decline in the physiological function of the brain in aging mice was improved [166]. Furthermore, the antineuroinflammation capacity of ganoderic acid A, the lanostane-type triterpenoid from *G*. *lucidum*, was assessed in lipopolysaccharide (LPS)-treated BV2 microglial cells. They found that the proliferation and activation of cells induced by LPS were markedly suppressed by ganoderic acid A. Furthermore, decreased production of IL-1β, IL-6, and TNF-α and enhanced brain-derived neurotrophic factor (BDNF) expression were detected in cells treated with ganoderic acid A. The antineuroinflammatory action of ganoderic acid A was also demonstrated by activation of the farnesoid-X-receptor, a transcriptional factor involved in neuroprotective functions [167]. In AD mice, ganoderic acid A has also been proven to attenuate neuroinflammation by modulating the imbalance of the Th17/Tregs [168]. Qi et al. further attempted to assess the protective potential of ganoderic acid A in the management of AD. Their results demonstrated that ganoderic acid A was capable of accelerating clearance of intracellular Aβ and alleviating cognitive impairment in mice with AD, which were attributed to the autophagy induced by ganoderic acid A via activation of Axl (a potential therapeutic target for the central nervous system) [169]. According to the results provided by Shen et al., it was speculated that the autophagy promoted by ganoderic acid A might be related to its regulation of PADI4, the peptidyl arginine deiminase type IV, which induces the autophagy in AD cells via the Akt/mTOR pathway [170].

Recently, the protective capacity of erinacines-enriched *H. erinaceus* mycelia against Parkinson’s disease (PD) [171], AD [172], spinocerebellar ataxia type 3 [173], age-related cognitive decline [174,175], etc. were described in a series of studies. In addition, the results of a study performed in 2020 showed that, compared with the control placebo group, patients with AD who received three capsules containing *H. erinaceus* mycelia (containing 5 mg/g erinacine A) showed significant neurocognition improvement [176]. The molecular mechanism of the positive activities of erinacine A may be attributed to their up-regulation of NGF expression and antineuroinflammatory and antiapoptosis effects. Following the detection of the expression of TNF-α and inducible nitric oxide synthase (iNOS), it was found that erinacine A treatment clearly decreased the expression of proinflammatory factors that activate glial cells and cell death induced by LPS. Furthermore, a neuroinflammation improvement effect was also observed in PD animal models treated with erinacine A [177]. Moreover, the iNOS/p38 MAPK- and TrkA/Erk1/2- mediated pathways were reported to participate in the neuroprotective effect of erinacine A [178,179].

Similarly, oral administration of another rare sesterterpene from *H. erinaceus*, called erinacine S, for 30 days alleviated the Aβ plaque phenomenon in APP/PS1 transgenic mice [180]. Additionally, the neuroprotective effects of other terpenes were identified, including cyrneines [181], scabronine M [182], sarcodonin [183], dictyophorines [184], lanostanoid triterpenes [185], neocyathins [164], striatoids [186], and (±)-Spiroganoapplanin A [187].

### 3.5. Vitamins

Recently, increasing evidence points to a correlation between vitamin deficiency and nervous system diseases, including AD and PD [188]. By comparing healthy volunteers with AD patients aged >64 years old, it was found that the serum vitamin D level of participants with AD was clearly lower [189]. The contributions of vitamins K_2_, A, and E to the pathogenesis of AD were also reported [190,191,192]. These findings suggest that vitamins are potential candidates for relieving NDs.

Mushrooms are generally considered natural sources of various vitamins, in particular vitamin D_2_ [193,194,195]. The fungi belonging to Basidiomycetes are rich in ergosterol, a type of sterol, which can be further metabolized into vitamin D_2_ after ultra violet (UV) irradiation. Huang et al. found that about 16.88 and 12.68 μg/g of Vitamin D_2_ and B_2_ were present in the commercially dried fruiting bodies of mushrooms in China, respectively [196]. As well as in the fruiting bodies, the vitamins were detected in mycelium harvested after liquid fermentation. *Pleurotus sapidus* was exposed to UV-B during liquid culture, and up to 365 μg vitamin D_2_ was determined in one gram of dried mycelium [197]. It is worth mentioning that mushroom powder containing 125–375 μg/g of vitamin D_2_ was requested to be designated a novel food in 2020 [198]. Recognizing the rich vitamin resources in mushrooms, ninety volunteers participated in the study by Stepien et al. to investigate whether supplementing mushrooms with vitamin D_2_ can improve vitamin D levels in adults. The data showed that intake of vitamin D2-enriched mushrooms markedly increased serum 25(OH)D_2_ concentration [199]. Therefore, vitamins from mushrooms show broad application prospects in the control of AD. The majority of studies have tried to increase the vitamin D_2_ content via ultraviolet irradiation of mushroom fruiting bodies. For example, the amount of vitamin D_2_ in the gills of shiitake mushrooms was increased more than 20 times after exposure to UV-B [200]. However, there is little literature focusing on the antiAD activities of vitamins in mushrooms, providing significant scope for future research. For example, Bennett and coworkers proved the function of button mushrooms (*Agaricus bisporus*) enriched with vitamin D_2_ on memory improvement in both wild and AD transgenic mice (APPswe/PS1dE9 transgenic mice) [201].

### 3.6. Nucleosides

In addition to the active components described above, nucleosides are another main ingredient of many mushrooms. Specifically, there are many studies on nucleosides in *Cordyceps*, and adenosine is considered the quality maker for the commercial application of *Cordyceps* extracts in Chinese Pharmacopoeia [202]. Various nucleosides, such as adenosine, cytidine, and uridine, have also been isolated because of their role in the regulation of various physiological processes (brain function, immunity, repair of gastrointestinal injury, etc.) [203].

In terms of neuroprotection, purine nucleosides from mushrooms may be promising candidate drugs for the future. The typical representative of these is cordycepin. Cordycepin, an analog of adenosine, is the natural constituent isolated from *Cordyceps*. It is reported that the amounts of cordycepin in the fruiting body and mycelial biomass of *C. militaris* stain CBS-132098 are 0.11%, and 0.182%, respectively [204]. Although the level of cordycepin is low, it exhibits a wide range of health-promoting potential applications, including neuroprotection. Evidence from the literature shows that cordycepin can regulate synaptic plasticity and overexcitation of the CA1 region, has antineuroinflammatory, antioxidation, and antiapoptosis effects, and can regulate mitochondrial dysfunction, thereby delaying the pathological changes caused by NDs [205,206]. For example, according to Olatunji et al., cordycepin alleviated the damage caused by 6-hydroxydopamine to PC12 cells, and this was attributed to its antioxidant properties and inhibition of cell apoptosis and mitochondrial dysfunction [207]. In another study, the therapeutic potential of cordycepin on neurological and cognitive impairments induced by intracerebral hemorrhage was proven via the regulation of oxidative stress [208]. Similarly, cordycepin decreased neural loss in mice with traumatic brain injuries. Furthermore, results from immunofluorescence staining, flow cytometry, and quantitative PCR showed that cordycepin treatment inhibited the proinflammatory microglia and macrophage in the cortex and striatum and increased the expression of anti-inflammatory microglia and macrophage. These data suggest that cordycepin alleviates long-term neuronal deficits [209]. Another similar study also documented that the potential mechanism of the neuroprotective effect of cordycepin is to relieve inflammatory response and nerve injury by inhibiting the release of NOD-like receptor thermal protein domain-associated protein 3 (NLRP3) inflammasomes [210].

Similarly, there has been extensive research concerning adenosine, another key purine nucleoside. Data showed that the viability of glutamate-induced PC12 cells pre-treated with adenosine derived from *C. cicadae* clearly increased, while ROS and Ca^2+^ levels in cells decreased. The authors mentioned that this protective property of adenosine was due to the regulation of the Bcl-2 family and a decrease in the levels of JNK, ERK, and phosphorylation of p38 [211]. In addition, the beneficial effect of other nucleosides and their derivatives from mushrooms (uridine [212], N^6^-substituted adenosine [213]) on neuroprotection was identified. Therefore, all studies indicate that mushroom nucleosides and their analogs may also be considered promising as therapeutic drugs for the treatment of AD.

### 3.7. Alkaloids

Alkaloids are nitrogen-containing compounds consisting of various subclasses. In addition to being widely present in plants, there is evidence to indicate that alkaloids are also important metabolites in mushrooms, with the most striking alkaloid being psilocybin derived from psilocybin mushrooms [214,215]. With the improvement in separation technology for natural products, various alkaloids were purified from mushrooms, some of which, such as corallocins and infractopicrin, have been found to have the potential to alleviate NDs [216]. The effect of the mushroom *Hericium coralloides*, famous in Chinese medicine, to promote NGF biosynthesis has been proven both in vitro and in vivo. Subsequently, the indole alkaloid corallocins A–C were isolated from this mushroom and were all capable of stimulating the expression of neurotrophin in human 1321N1 astrocytes [15,217]. Another pyrrole alkaloid (inotopyrrole B) was isolated from the mushroom *Phelbopus portentosus* by Sun et al., and its ability of neuroprotection was proven in human neuroblastoma SH-SY5Y cells damaged with H_2_O_2_ [218]. Ryu et al. reported that isohericerinol A, a new isoindolinone derivative isolated from *H. erinaceus*, accelerated the synthesis of NGF and further stimulated the neurite outgrowth, suggesting that this substance may be able to alleviate AD symptoms. Western blot detection revealed that the expression of neurotrophins, including brain-derived neurotrophic factor (BDNF) and synaptophysin, were both enhanced in C6-N2a neuronal cells treated with isohericerinol A [219]. In the study performed by Lee et al., dictyoquinazol A–C isolated from *D. indusiata* was capable of reducing the risk of neurotoxicity and cell death of primary mouse cortical neurons induced by glutamate and N-methyl-D-aspartate at lower concentrations [220]. Additionally, there have been reports on the ability of other mushroom alkaloids, such as ganocochlearine A, infractopicrin, and 10-hydroxy-infractopicrin, to alleviate NDs [221,222]. Because of the neuroprotective potential of these compounds, the synthesis of isohericerinol, dictyoquinazol, and related derivatives has been continuously explored [223,224]. However, mushrooms remain the main sources of these compounds, and it is particularly important to explore their neuroprotective mechanisms in vivo.

### 3.8. Sterols

Compared with other components, there are few reports on the neuroprotective effects of mushroom sterols. In a study by Zhao et al., a novel sterol (matsutakone) and norsteroid (matsutoic acid) were successfully obtained from the edible mushroom *Tricholoma matsutake*, which is a delicious mushroom used in China. Furthermore, the AChE-inhibiting actions of these two compounds at a concentration of 50 μM were observed [225]. Ergosterol peroxide, a lipid-soluble steroid derivative, was isolated from a variety of mushrooms (*G. lucidum*, *I. obliquus*, *P. ostreatus*, etc.), and the weak ability of ergosterol peroxide from *H. erinaceus* has been demonstrated in PC12 cells [226,227,228,229]. However, although several in vitro experiments have confirmed the neuroprotective effects of mushroom sterols, further work is needed to assess the possibility and mechanisms of the aforementioned sterols in the treatment of AD in vivo, as well as the identification of new mushroom sterols.

### 3.9. Other Constituents

In addition to the above components, other constituents of mushrooms, such as essential oils, benzofuran derivatives, and cerebroside-A, were reported to have potential therapeutic activities for NDs, and these are also worthy of further exploration [230,231,232].

## 4. Conclusions and Future Research Prospects

Diverse mushrooms can be regarded as treasure troves of active compounds which have been widely used for both medical and food purposes. There are many research articles concerning the antiAD activity and molecular mechanisms of mushrooms. This review summarizes information regarding the therapeutic potential of the diverse metabolites from various mushrooms for neuroprotection, with particular emphasis on AD. Overall, many types of mushrooms have shown beneficial effects in alleviating AD, and these are attributed to their large number of constituents, such as polysaccharides, phenols, and peptides. The mechanisms involved in the antiAD activities of mushroom metabolites include antioxidant and antineuroinflammatory activity, apoptosis inhibition, and stimulation of neurite outgrowth, etc. The information collected in this review suggests that mushrooms and their metabolites have broad application prospects in the management of AD as well as NDs more widely. However, more work is required regarding the purification of active compounds, elucidation of their antiAD capacity and mechanisms in vivo, toxicity, improvement of their bioavailability and production, and development of related products. Significant work remains to be carried out to explore the vast untapped resources of mushrooms, including their cultivation and their medicinal and edible potential.

## Data Availability

Not applicable.

## Abbreviations

Alzheimer’s disease (AD); Neurodegenerative diseases (NDs); Parkinson’s disease (PD); Central nervous system (CNS); Amyloid beta peptide (Aβ); Neurofibrillary tangles (NFTs); Reactive oxygen species (ROS); Reactive nitrogen species (RNS); Superoxide dismutase (SOD); Programmed cell death (PCD); Jun N-terminal kinase (JNK); Brain-derived neurotrophic factor (BDNF); Nerve growth factor (NGF); Lipopolysaccharide (LPS); 1-methyl-4-phenyl-1,2,3,6-tetrahydropyridine (MPTP); NOD-like receptor pyrin domain containing three (NLRP3); Dynamin-related protein-1 (Drp1); Dry weight (DW); Hydrogen peroxide (H_2_O_2_); Mitogen-activated protein kinase (MAPK); Hippocampal neuron cell line (HT22); Pheochromocytoma cells (PC12 cells); Mouse microglial cells (BV-2 cells); Inducible Nitric Oxide Synthase (iNOS); Nuclear factor-κ-gene binding (NF-κB); Toll like receptor (TLR); Extracellular-signal regulated kinases (ERKs); Protein kinase B (Akt); Mammalian Target of Rapamycin (mTOR).
